# Immunotherapy‐Resistant Neuropathic Pain and Fatigue Predict Quality‐of‐Life in Contactin‐Associated Protein‐Like 2 Antibody Disease

**DOI:** 10.1002/ana.27177

**Published:** 2025-01-18

**Authors:** Bryan Ceronie, Christine Strippel, Christopher Uy, Sofija Paneva, Mateusz Makuch, Babak Soleimani, Sanchit Turaga, Sophie Binks, Sudarshini Ramanathan, Sophia Michael, James Varley, Ava Easton, Andreas Themistocleous, John Dawes, David L. Bennett, Anushka Irani, Adam E. Handel, Sarosh R. Irani

**Affiliations:** ^1^ Oxford Autoimmune Neurology Group, Nuffield Department of Clinical Neuroscience Oxford UK; ^2^ Department of Neurology & Neuroimmunology University of British Columbia Vancouver British Columbia Canada; ^3^ Nuffield Department of Orthopedics, Rheumatology and Musculoskeletal Services Oxford UK; ^4^ Department of Neurology, John Radcliffe Hospital Oxford UK; ^5^ Translational Neuroimmunology Group, Faculty of Medicine and Health University of Sydney Sydney New South Wales Australia; ^6^ Department of Neurology, Concord Hospital Sydney New South Wales Australia; ^7^ Department of Neurology, Queen Elizabeth Hospital Birmingham UK; ^8^ Department of Brain Sciences, Charing Cross Hospital Imperial College London London UK; ^9^ Encephalitis International North Yorkshire UK; ^10^ Department of Clinical Infection, Microbiology & Immunology, Institute of Infection, Veterinary and Ecological Sciences University of Liverpool Liverpool UK; ^11^ Neural Injury Group, Nuffield Department of Clinical Neuroscience Oxford UK; ^12^ Department of Neurosciences, Mayo Clinic Jacksonville Florida USA; ^13^ Department of Neurology, Mayo Clinic Jacksonville Florida USA

## Abstract

The long‐term clinical outcomes and associated prognostic factors in contactin‐associated protein‐like 2 (CASPR2)‐antibody diseases are unknown. A total of 75 participants with CASPR2 antibodies were longitudinally assessed for disability, quality‐of‐life, and chronic pain. Although most symptoms improved within 6 months of treatment, neuropathic pain and fatigue were the most immunotherapy refractory, and persisted for up to 6 years. Furthermore, these two factors—but not CASPR2 antibody levels or subclasses—independently predicted worse disability and quality‐of‐life at 24 months. Quality‐of‐life varied widely for any given modified Rankin Scale score, indicating a divergence between patient and clinician assessed outcomes. Further work should study the relative importance of these measures, and the immunopathogenesis underlying intractable symptoms. ANN NEUROL 2025;97:521–528

Contactin‐associated protein‐like 2 (CASPR2)‐antibody diseases present with myriad features and are often classified as 3 main syndromes: limbic encephalitis (LE), Morvan's syndrome (MoS), or peripheral nerve hyperexcitability (PNH).^1^, ^2^, ^3^, ^4^ Neuropathic pain (NeP) is present in 30–50% of cases, and can be associated across these 3 syndromes.^5^, ^6^, ^7^

Although the presenting features of CASPR2‐antibody diseases have been well‐characterized, the long‐term clinical outcomes, including quality of life (QoL) measures, have received limited attention. Elucidating factors associated with disability and QoL allows for improved clinical decision‐making and more accurate prognostication of disease trajectories. In search of such predictive factors, we describe and quantify the long‐term symptom burden of patients with CASPR2‐antibody disease, disability, and patient‐rated QoL, and characterize clinical and biological factors that may predict these outcomes.

## Methods

### Study Design

The study was approved by Leeds East Research Ethics Committee (16/YH/0013), and all participants enrolled with written, informed consent. Participants were selected from the Oxford Autoimmune Neurology Group and a previously published MoS study database^2^ with CASPR2 immunoglobulin G (IgG) detected on live cell‐based assays at a stringent serum titer of >1:400. Patients and relatives underwent telephone interviews in conjunction with medical record review to ascertain the presence or absence of symptoms, therapy administration, tumor associations, and investigations. NeP was identified based on clinical features in accordance with the Special Interest Group on Neuropathic Pain (NeupSIG) grading system.^8^ Outcome measures, including the modified Rankin Scale (mRS), EuroQol 5D QoL visual analog scale (EQ5DVAS),^9^ and chronic pain grading scale (CPGS),^10^ were all retrospectively assessed by recall of peak symptom burden, thereafter at 6 months, and then yearly for up to 6 years. Frequently identified cancers and benign prostatic hypertrophy (BPH) were compared to the age‐standardized rate in the general population based on disease registry data,^11^, ^12^, ^13^ using prevalence to reduce bias of detecting incidental lesions through routine screening. Unbiased hierarchical clustering used Euclidean distance and complete linkage to validate clinical syndromic classifications.

CASPR2‐IgG, IgG1, and IgG4 testing methods (similar to those used by Thompson et al.^14^) are described in supporting information.

### Statistical Analysis

Data visualization and statistical analysis were performed on Prism (v10; GraphPad, San Diego, CA, USA) and R Studio (v2023.06.1 + 524, Posit Software, PBC, for packages; Boston, MA, USA; Table S3). As data were non‐normally distributed, univariate testing was performed by Kruskal–Wallis and Mann–Whitney tests with bootstrapped medians and standard errors, curve comparison with a likelihood ratio test of polynomial linear mixed effects models, correlation by Spearman's test, and Firth multiple logistic regression using a “bad” outcome of below (for EQ5DVAS) or above (for mRS and CPGS) the median for a given time point. Bonferroni correction was used for univariate multiple comparisons.

## Results

### Patient Characteristics

**FIGURE 1:**
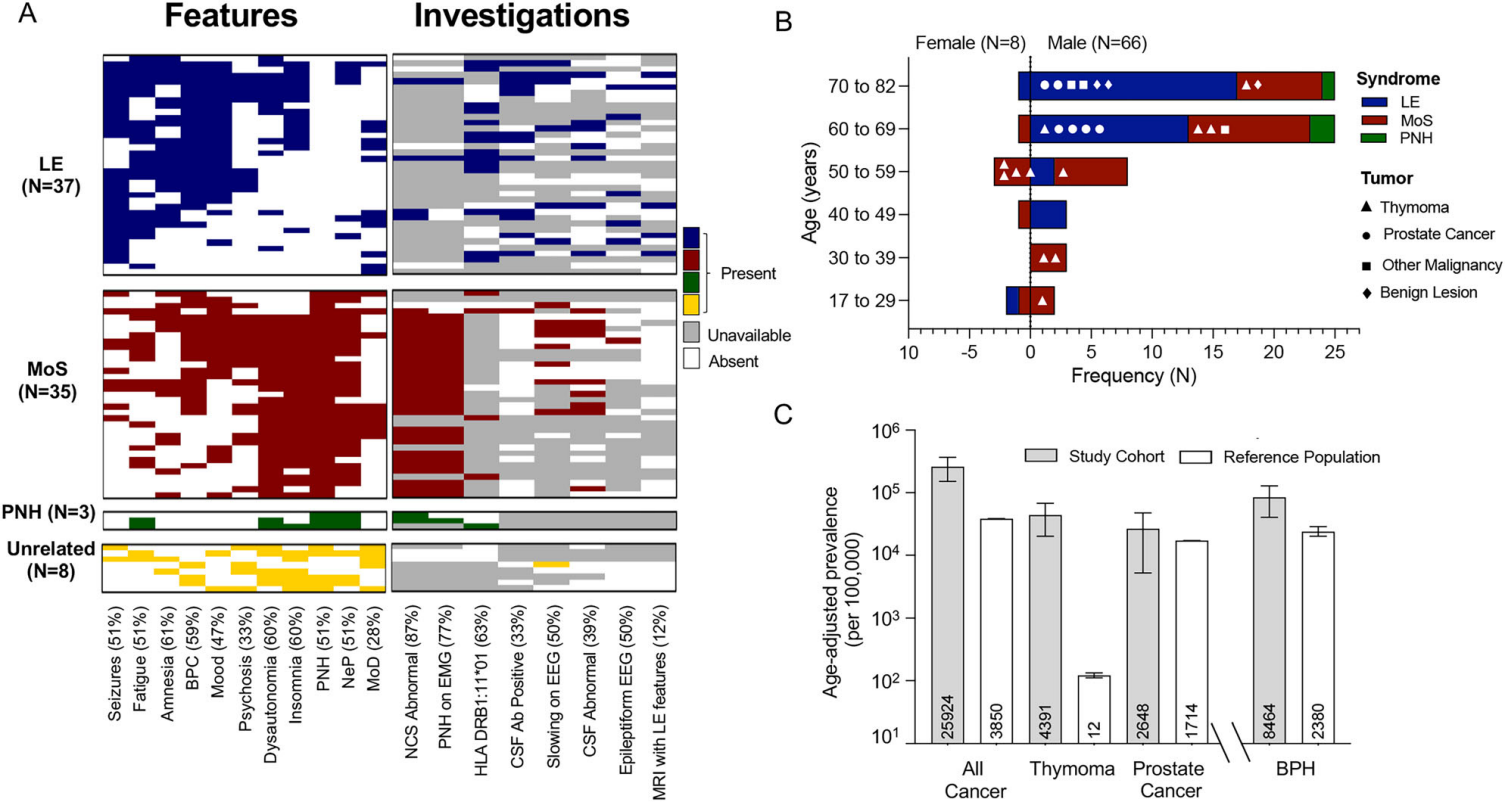
Demographics, tumor association, and clinical features by syndrome. (A) Of 83 participants with contactin‐associated protein‐like 2 (CASPR2) antibodies, 75 were diagnosed with clinically‐related CASPR2‐antibody disease (8 had an unrelated clinical syndrome): limbic encephalitis (LE; *n* = 37), Morvan's Syndrome (MoS; *n* = 35), and peripheral nerve hyperexcitability (PNH; *n* = 3). (B) Of *n* = 75, 66 were male, and showed a median age of 66 years (range 17–82 years). Whole‐body imaging revealed 25 lesions: 12 malignant thymomas, 6 prostate cancers, 3 other cancers (small cell lung, bowel, and Merkel cell carcinoma), and 3 benign lesions. (C) Based on disease registry data, the age‐adjusted prevalence of all cancers, prostate cancer, and thymoma were overrepresented in our cohort compared with the general population. Similarly, the rate of benign prostatic hypertrophy (BPH) was overrepresented compared with the male general population. BPC = behavioral/personality change; CSF = cerebrospinal fluid; EEG = electroencephalography; EMG = electromyography; HLA = human leukocyte antigen; LE = limbic encephalitis; MoD = movement disorder; Mood = mood disturbance; MoS = Morvan's syndrome; MRI = magnetic resonance imaging; NeP = neuropathic pain; NCS = nerve conduction studies; PNH = peripheral nerve hyperexcitability. [Color figure can be viewed at www.annalsofneurology.org]

A total of 83 patients with CASPR2‐IgGs were identified: 8 of 83 were excluded due to an alternative clinical diagnosis made by their neurologist (Table S1). The remaining 75 patients were included as definite CASPR2‐antibody diseases (Fig. 1A). The median duration of follow‐up was 4.25 years (range 0–26 years), with 23 of 75 followed up at 6 years (Fig. S1A). There were 66 males (88%), and the overall median age was 66 (range 17–82; Fig. 1B). There were 6 deaths during the study period (Table S2), most unrelated to CASPR2 antibody disease or immunotherapy. The median time from onset to symptom peak was 4.4 weeks (range 0–165 weeks), and from onset to treatment was 10.8 weeks (range 0–178 weeks); 34.7% of cases reached symptom peak after 3 months (Fig. S1A,B).

### Disease Categories and Tumor Associations

From 75 patients, clinical judgement using symptoms at onset classified 37 with LE, 35 as MoS, and 3 as PNH (Fig. 1A). Unbiased hierarchical clustering accurately aligned with this—creating two distinct syndromic groups (LE and MoS‐PNH) in all but 3 cases, who presented with overlapping features of both LE and MoS. No distinction was made between MoS and PNH (Fig. S1C).

A total of 25 (33%) tumors were identified: 13 thymomas, 6 prostate cancers, 3 other malignancies (non‐small cell lung cancer, colon cancer, and Merkel cell carcinoma), and 3 benign lesions (thymic hyperplasia, thymic cyst, and a pancreatic cyst; Fig. 1B). A total of 12 of 13 (92%) thymomas were in the MoS group, whereas 6 of 6 (100%) prostate cancers were in the LE group. An additional 14 patients (21% of males) had BPH. By comparison with the general population, disease prevalence in the study cohort was higher for all cancers (age‐standardized rate ± SE 25,924 ± 5,527 vs 3,850 ± 2.7 per 100,000 persons at risk), prostate cancer (2,648 ± 1,081 vs 1,714 ± 2.6), thymoma (4,391 ± 1,217 vs 12 ± 0.58), and BPH (8,464 ± 2,262 vs 2,420 ± 217, Fig. 1C).

### NeP and Fatigue Are Prominent Long‐Term Features and Associate with Disability and QoL

**FIGURE 2:**
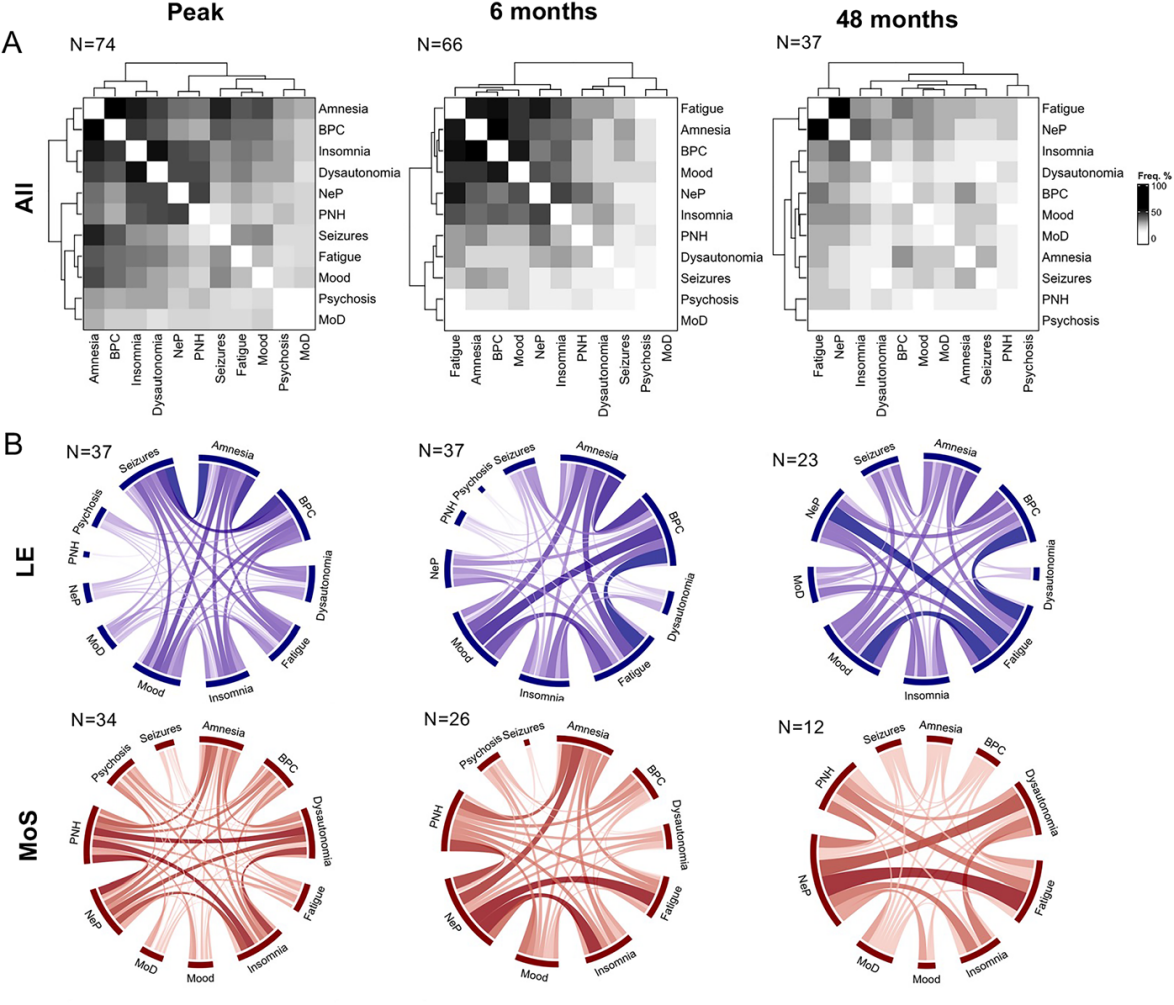
Relative symptom frequencies over time. (A) The relative frequency of symptom pairs was evaluated in all patients at the peak of disease, 6 months and 48 months. At peak, amnesia, behavioral/personality change (BPC), insomnia and dysautonomia co‐occurred most frequently, followed by neuropathic pain (NeP) and fatigue. At 6 months, NeP persisted, and fatigue became more prominent. By 48 months, as most other symptoms improved, persistent NeP and fatigue emerged as the most prominent symptom pair. (B) The frequency of co‐occurrent symptoms was evaluated in the two main syndrome groups, limbic encephalitis (LE) and Morvan's syndrome (MoS). At peak in the LE group, seizures and BPC were most frequent, whereas the MoS group showed prominent co‐occurrence of NeP, peripheral nerve hyperexcitability (PNH), dysautonomia, and insomnia. At 6 months, BPC, mood disorder, and fatigue were most prominent in the LE group, whereas in the MoS group, NeP, fatigue, and insomnia co‐occurred most frequently. By 48 months, as most other symptoms improved, NeP and fatigue were the most prominent residual symptoms in both the LE and MoS groups. MoD = movement disorder. [Color figure can be viewed at www.annalsofneurology.org]

**FIGURE 3:**
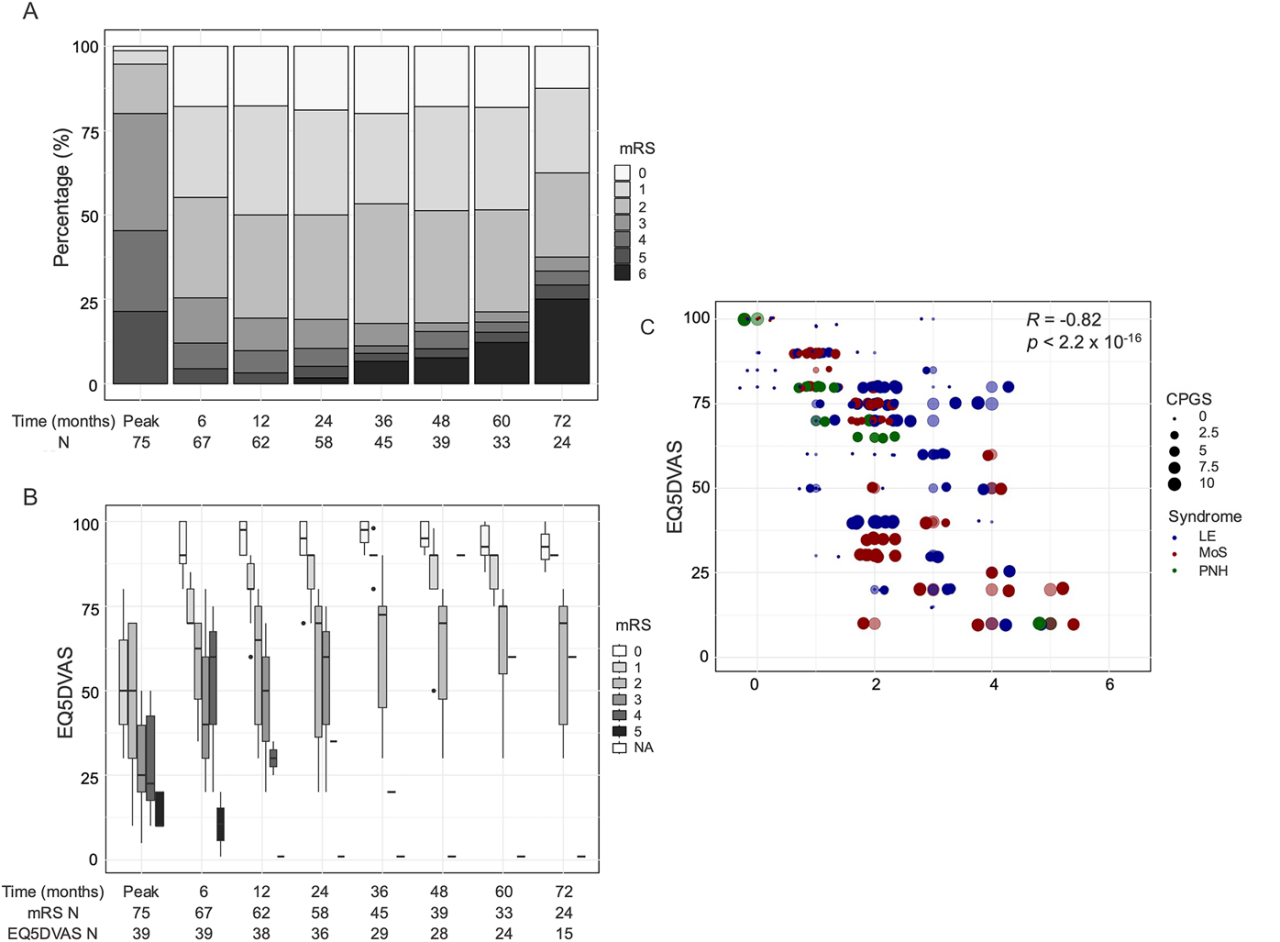
Longitudinal outcome measures in contactin‐associated protein‐like 2 (CASPR2) antibody diseases. (A) Modified Rankin Scale (mRS) scores at the peak of disease through to 72 months. (B) EuroQol 5D Visual Analog Scale (EQ5DVAS) variation with mRS and over time (to 72 months). Deaths were carried forward such that there were 6 of 24 (25%) at 72 months. (C) Across all time points, mRS correlated with EQ5DVAS (*R* = −0.82, *p* < 2.2 × 10^−16^), and showed variation with pain severity (as measured by Chronic Pain Grading Scale [CPGS]). IT = immunotherapy; LE = limbic encephalitis; MoS = Morvan's Syndrome; mRS = modified Rankin Scale; NeP = neuropathic pain; PNH = peripheral nerve hyperexcitability; QoL = quality of life. [Color figure can be viewed at www.annalsofneurology.org]

At symptom peak, dysautonomia, behavioral features, amnesia, and insomnia were the most frequent symptoms. However, by 6 months and more strikingly at 48 months, as the relative frequencies of other symptoms declined with treatment, NeP and fatigue became the most prominent, typically co‐occurrent, symptoms in the two main groups, LE and MoS (Fig. 2). This did not appear biased by duration of follow‐up, as those followed up to 48 months had similar presentations to the overall cohort (Fig. S3), and duration of follow‐up was not associated with differences in mRS at nadir or overall treatment strategies (Fig. S4). Over this time, and with longer follow‐up, both median mRS (Fig. 3A) and EQ5DVAS (Fig. 3B) improved, although most improvements were noted within the first 6 months after treatments. Notably, at all timepoints, EQ5DVAS scores showed large variance for any given mRS (Fig. 3B), indicating that individual EQ5DVAS values were associated with multiple mRS scores, and vice versa. Nevertheless, mRS correlated well with EQ5DVAS (*R* = −0.82, *p* < 2.2 × 10^−16^; Fig. 3C) and pain severity (CPGS; *R* = −0.5, *p* = 8.4 × 10^−11^; Fig. S2A). Yet, within this, patients with the least pain showed some of the best outcomes (lower mRS and higher EQ5DVAS; Fig. 3C). Given that 46 of 49 (94%) of the pain was neuropathic, this suggested NeP may represent an important driver of disability and QoL.

### NeP and Fatigue Do Not Respond to Immunotherapy and Are Associated with Poor Outcomes

**FIGURE 4:**
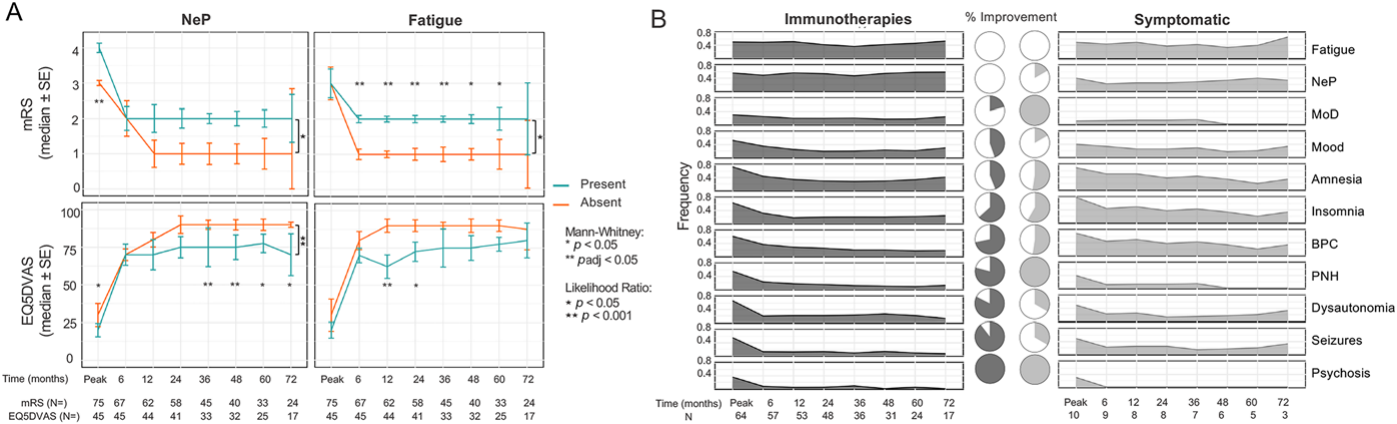
The impact of neuropathic pain (NeP) and fatigue impact on longitudinal outcome measures and response to immunotherapy. (A) NeP and fatigue both associated with higher modified Rankin Scale (mRS) and lower EuroQol 5D Visual Analog Scale (EQ5DVAS) scores. Unadjusted (*) and adjusted (**) *p* values (<0.05) highlight statistically significant differences in univariate tests, and ^★^ *p* < 0.05 and ^★★^ *p* < 0.001 indicate statistically significant differences between curves (fitted with polynomial linear mixed effects model). (B) Symptom prevalence over time in groups divided by those treated with immunotherapy (*n* = 64) and only symptomatic therapies (*n* = 10). BPC = behavioral or personality change; CASPR2 = contactin‐associated protein‐like 2; CPGS = Chronic Pain Grading Scale; IT = immunotherapy; MoD = movement disorder; Mood = mood disturbance; NeP = neuropathic pain; PNH = peripheral nerve hyperexcitability; QoL = quality of life. [Color figure can be viewed at www.annalsofneurology.org]

**FIGURE 5:**
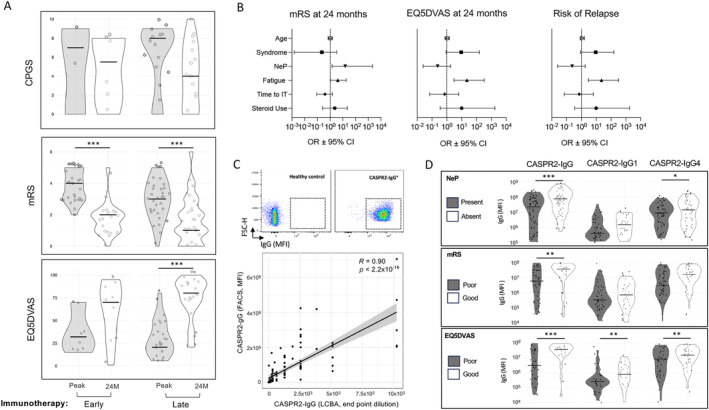
Clinical and biological factors associated 24‐month outcomes and relapses. (A) Groups divided by early (<12 weeks) or late (>12 weeks) immunotherapy have Chronic Pain Grading Scale (CPGS), modified Rankin Scale (mRS), and EuroQol 5D Visual Analog Scale (EQ5DVAS) scores compared between the peak and 24 months. Multiple comparison adjusted significance observed (***Padj < 0.01). (B) Multiple logistic regression of selected clinical predictors mRS or EQ5DVAS at 24 months, or relapse at any point. A worse outcome was defined as a value above (for mRS) or below (for EQ5DVAS) the median for a given time point. Neuropathic pain (NeP) at onset (OR 15.5, 95% CI 1.4–2,173) and fatigue at onset (OR 4, 95%CI 1.1–18.5) were associated with worse mRS outcomes at 24 months and fatigue in EQ5DVAS at 24 months (OR 6.2, 95%CI 1.1–50.1), and fatigue also predicted an increased risk of relapse (OR 22.4, 95%CI 3–327.4). (C) Representative flow cytometry plots (top two panels) showing immunoglobulin G (IgG) binding (IgG mean fluorescence intensity [MFI], *x*‐axis) to CASPR2‐expressing human embryonic kidney cells in sera from healthy controls (left) and CASPR2‐IgG patients (right). Dotted gate quantifies CASPR2‐reactive IgGs by flow cytometry (IgG‐FACS). Lower: CASPR2‐IgG by FACS correlates well with CASPR2‐IgG levels by cell‐based assay (CBA) endpoint dilution (*R* = 0.90; *p* < 2.2 × 10^−16^) with wide variation in FACS data per endpoint dilution. (D) CASPR2‐IgG, ‐IgG1, and ‐IgG4 quantification by FACS. Those with NeP showed lower with median CASPR2‐IgG (Padj < 0.01); poor mRS and EQ5DVAS outcomes at 24 months were associated with lower median CASPR2‐IgG (Padj < 0.05), and ED5DVAS with lower CASPR2‐IgG1 (Padj < 0.05) and lower CASPR2‐IgG4 levels (Padj < 0.05). *p < 0.05; **Padj < 0.05; ***Padj < 0.01. [Color figure can be viewed at www.annalsofneurology.org]

Division of patients with and without NeP or fatigue at onset revealed consistent differences in their longitudinal mRS and EQ5DVAS trajectories (Fig. 4A). This was confirmed with linear regression: NeP at onset was associated with higher mRS (G^2^ 11.13, *p* = 0.025) and lower EQ5DVAS (G^2^ 10.30, *p* = 0.035) trajectories; with similar findings for fatigue at onset (G^2^ 20.38, *p* = 0.00042 for mRS; G^2^ 9.19, *p* = 0.056 for EQ5DVAS). Furthermore, although NeP and fatigue were present at the peak in a subset of patients, in contrast to other symptoms whose frequency reduced with immunotherapies, and sometimes with symptomatic therapies alone, the proportion of patients with fatigue or NeP did not reduce in response to either intervention (Fig. 4B). In fact, even early (<12 weeks) immunotherapy initiation was not associated with improvements in CPGS between the peak and 24 months, by contrast to median mRS (*p* = 3.3 × 10^−7^ and *p* = 0.00016, respectively) and EQ5DVAS (*p* = 1.65 × 10^−6^ for late initiation; Fig. 5A). Hence, again, immunotherapy was not associated with improvements in pain outcomes.

### NeP and Fatigue Predict Worse Outcomes and Risk of Relapse

Next, NeP and fatigue were incorporated alongside other clinical predictors into a multiple logistic regression model. Although age, syndrome, time to immunotherapy, and steroid use were not associated with any of the outcomes, NeP predicted a worse mRS (OR 15.5, 95% CI 1.4–2,173), and fatigue predicted both worse mRS (OR 4, 95% CI 1.1–18.5) and EQ5DVAS (OR 6.2, 95% CI 1.1–50.1) at 24 months.

As our cohort were collected over many years, the duration of follow‐up was studied as a potential confounder (Fig. S4). However, this was not associated with mRS at peak, nor types of immunotherapies (Fig. S4A–C), but consistent with increasing disease recognition over time, was associated with longer time to immunotherapy (Fig. S4D). Nevertheless, regression modeling confirmed that only mRS at nadir predicted a worse outcome at 24 months, with no significant interactions (Fig. S4E). Interestingly, fatigue was the only factor associated with increased risk of relapse (OR 22.4, 95% CI 3–327.4; Fig. 5B, Table S4).

### NeP and Fatigue Are Associated with Differences in IgG Subclasses

Finally, we asked if NeP, disability, or QoL correlated with total or subclass‐specific CASPR2 antibody levels, as measured by a highly quantitative flow cytometry based live cell‐based assay (Fig. 3C). Surprisingly, all of CASPR2‐specific IgG, IgG1, and ‐IgG4 were consistently lower in patients with NeP, and showed worse 24‐month outcomes (Fig. 3D; Fig. S2B).

## Discussion

In a large cohort of patients with CASPR2 antibody disease, the present study focused on long‐term clinical outcomes and their predictors. Although most symptoms improved markedly by 12 months after treatment initiation, NeP and fatigue appeared as prominent features after 6 months and persisted at 4 years in up to 60% of patients, despite adequate immunotherapies. Furthermore, in contrast to more predictable associations with disability, QoL and relapses, NeP and fatigue were the only factors associated with poorer outcomes, emphasizing these as key drivers of long‐term disease burden. Their association with lower CASPR2‐IgG/IgG1/IgG4 levels highlights the need for more nuanced studies to appreciate mechanisms by which pathogenic CASPR2 antibodies induce NeP,^15^ and, hence, offer more precision treatments for these important QoL‐determining features.

Patients with neuropathic pain plus LGI1 or CASPR2 antibodies often show reduced intraepidermal fiber density on skin biopsy (in this study, all 3/75 tested were abnormal).^6^, ^7^ Assuming this is a dominant mechanism of their pain, and clearly pain responds poorly to immunotherapies, it may be that mechanisms to modulate CASPR2 expression in peripheral nerves is a more tractable therapeutic avenue. Yet, sadly, it may be that small fiber damage is sustained and irreparable.

Our data also highlight several likely generalizable observations. First, although they showed overall good correlations between outcome measures, each (clinician‐rated) mRS point showed substantial overlaps and variance in EQ5DVAS, indicating patient‐rated scores offer greater dynamic ranges and meaning as outcome measurements. Given that mRS 0–2 is typically classified as a “good” outcome, yet shows huge variability in EQ5DVAS, our observation calls for QoL measures to replace mRS in future studies of CASPR2 antibody disease, and likely other forms of autoimmune encephalitis.^16^, ^17^ Although no tailored patient‐reported outcomes measures for autoimmune encephalitis currently exist,^18^ it is clear that over‐reliance on a disability score developed for stroke fails to capture the myriad residual symptoms that burden patients.^19^

Next, although thymoma is a well‐recognized association of CASPR2 antibody diseases, it was surprising to observe overrepresentation of prostate cancer and BPH compared with the general population. Although this may be biased by increased detection of incidental lesions through routine cancer screening in our patients, CASPR2 mRNA is detectable in the nervous system and prostate.^20^, ^21^, ^22^ This raises the possibility that prostate antigens might activate circulating CASPR2‐reactive B cells, and may begin to account for the phenotype of almost exclusively older men, a highly atypical demographic for any autoimmune disease. Interestingly, the rate of prostate cancer has been reported as 5.1% in LGI1 antibody encephalitis,^23^ which is higher than the unadjusted population prevalence.^24^ Also, we observed that patients with CASPR2 antibody disease often fall outside of diagnostic criteria^25^ for autoimmune encephalitis, as many only present after 3 months (as previously reported^4^), yet late immunotherapy remains effective in improving QoL and mRS. Hence, our data suggest immunotherapy should be trialed, even in later presentations, which may benefit symptoms other than NeP and fatigue. Although it is not clear why fatigue may be a predictor of relapse, it may represent a more severe overall phenotype, and thus a lower likelihood of being immunotherapy‐responsive.

Limitations of this study include its retrospective design, with potential for recall bias in assessing symptoms and clinical outcome measures. As a quaternary referral center, there may also have been a referral bias of patients with atypical demographics or clinical presentations. A proportion of the cohort was lost to follow‐up, which could also result in a selection toward more severe, treatment‐refractory phenotypes, but our analyses of the 48‐month followed‐up cohort suggested this was an actual bias herein. Similarly, varied follow‐up durations were not a confounder in affecting outcomes. Finally, we did not specifically assess other quantifiable symptom‐specific outcome measures, such as fatigue or cognition, an aim for future studies.

CASPR2 antibody disease now joins other autoimmune encephalopathies and systemic rheumatological diseases, where pain and/or fatigue are often persistent and debilitating features, and should be assessed routinely in the appropriate setting^16^, ^17^, ^26^ Understanding neuroimmune mechanisms underlying this phenomenon are now required to improve patient care and quality of life.

## Author Contributions

C.U., A.I., A.E.H., and S.R.I. contributed to conception and design of the study. B.C., C.S., C.U., S.P., M.M., B.S., S.T., S.B., S.M., and J.V. contributed to acquisition and analysis of the data. B.C. and C.S. contributed to drafting of the text and editing of the figures.

## Potential Conflicts of Interest

S.B. is named on a patent application entitled “Diagnostic Strategy to improve specificity of CASPR2 antibody detection” (TBA / BB Ref. JA94536P.GBA)”. D.L.B. has a patent application “A method for the treatment or prevention of pain, or excessive neuronal activity, or epilepsy”, Application No. 16/337,428. S.R.I. receives licensed royalties on patent application WO/2010/046716 entitled “Neurological Autoimmune Disorders,” and has filed two other patents entitled “Diagnostic method and therapy” (WO2019211633 and US app 17/051,930; PCT application WO202189788A1) and “Biomarkers” (WO202189788A1, US App 18/279,624; PCT/GB2022/050614).

## Supporting information


**Supplementary Table S1.** List of unrelated cases excluded from the study. All cases were seen by an experienced consultant neurologist and their CASPR2‐antibody seropositivity was deemed to be irrelevant to the presenting clinical syndrome. CASPR2 = contactin‐associated protein‐like 2; CSF = cerebrospinal fluid; EMG = electromyography; LCBA = live cell‐based assay; MRI = magnetic resonance imaging; VGKC = voltage‐gated potassium channel.

**Supplementary Table S2.** Causes of death during the study. Relationships to either CASPR2‐antibody encephalitis or the consequences of immunotherapy. CASPR2 = contactin‐associated protein‐like 2; IT = immunotherapy; IVIG = intravenous immunoglobulin; PLEX = plasma exchange; SUDEP = sudden unexplained death in epilepsy.

**Supplementary Table S3.** List of R packages and corresponding citations.

**Supplementary Table S4.** Numbers of relapses and absolute risk for (A) first relapse and (B) all relapses, as in some cases patients had more than one relapse. Relapses were most frequent in the first 24 months from the peak of the disease and were more common in those with fatigue at onset than those without.

**Supplementary Figure S1.** (A) Median duration of follow‐up was 4.25 years (range 0–26), with follow‐up declining from 75 cases at peak to 24 cases at 6 years. (B) Median time from onset to the peak of disease symptoms was 4.42 weeks (range 0–165), median time from onset to treatment was 10.78 weeks (range 0–178). However, 34.7% of cases reached peak after 3 months. (C) To validate our syndromic classification, we used an unbiased hierarchical clustering method with Euclidean distance and complete linkage. Clustering accurately differentiated LE and MoS/PNH patients into 2 distinct groups in all but all but 3 cases, who presented with features of both. It did not differentiate between MoS and PNH. BPC = behavioural/personality change; CASPR2 = contactin‐associated protein‐like 2; LE = limbic encephalitis; MoD = movement disorder; Mood = mood disturbance; MoS = Morvan's Syndrome; NeP = neuropathic pain; PNH = peripheral nerve hyperexcitability.

**Supplementary Figure S2.** (A) CPGS negatively correlated with mRS (*R* = −0.5, *p* = 8.4 × 10^−11^) and EQ5DVAS (*R* = −0.47, *p* = 5.9 × 10^−11^) As 46/49 (94%) of pain symptoms were neuropathic, this strongly suggested NeP as an important driver of disability and QoL. (B) EPT correlated strongly with IgG1 (*R* = 0.57, *p* = 1.5 × 10^−8^) and IgG4 (*R* = 0.85, *p* < 2.2 × 10^−16^) and. Top panels show representative flow cytometry plots of gating strategy for IgG quantification: HEK23T cells that had been transfected with CASPR2‐eGFP were incubated with patient plasma/serum then an anti‐IgG, IgG1 or IgG4 secondary antibody. Cells were then gated on live singlet GFP‐expressing events and the mean fluorescence intensity calculated on the basis of the geometric mean of the secondary fluorophore. This was then quantified as IgG based on the antibody binding capacity of anti‐IgG standards. Bottom panels show representative positive patient samples and negative healthy controls for different MFIs. (C) Violin plots showing the association between fatigue and CASPR2‐IgG, IgG1 and IgG4 subclasses. There were no significant differences between groups in any of the subclasses. CASPR2 = contactin‐associated protein‐like 2; CPGS = chronic pain grading scale; eGFP = enhanced green fluorescent protein; EPT = end point titre; HEK293T = human embryonic kidney 293T cell; IgG = immunoglobulin G; MFI = mean fluorescence intensity; NeP = neuropathic pain.

**Supplementary Figure S3.** (A) Heatmaps of relative frequency at peak for the subgroup of patients under follow‐up at peak (*n* = 74), 6 months (*n* = 66) and 48 months (*n*= 37). (B) Circos plots showing the co‐occurrence of symptoms divided by syndrome for those still under follow‐up at peak, 6 and 48 months. For LE this was 37, 37 and 23 patients respectively, and for MoS this was 34, 26, and 12 patients, respectively. BPC = behavioural/personality change; CASPR2 = contactin‐associated protein‐like 2; LE = limbic encephalitis; MoD: =movement disorder; Mood = mood disturbance; MoS = Morvan's Syndrome; NeP = neuropathic pain; PNH = peripheral nerve hyperexcitability.

**Supplementary Figure S4.** (A) Violin plots showing mRS at peak symptom burden with length of follow‐up divided into terciles: <2.8, 2.8–5.6 and <5.6 years. Kruskall‐Wallis analysis reveal no significant differences in mRS at peak between the groups. (B) Box plots showing time to immunotherapy in weeks with duration of follow‐up divided into terciles. Longer duration of follow‐up with was significantly longer time to immunotherapy between <2.8 and 2.8–5.6 years (median ± SE 1.84 ± 0.21 vs 4.25 ± 0.18, *p* = 0.015) and between <2.8 and >5.6 years (1.84 ± 0.21 vs 8.54 ± 0.88, *p* = 0.0036). (C) Mosaic plot showing frequencies treatment modalities divided into terciles. Chi‐Squared analysis revealed no significant difference between the groups. (D) Box plots showing time to immunotherapy divided by mRS at peak. Kruskal‐Wallis analysis revealed no significant differences between the groups. (E) Variables were combined into a multiple logistic regression model using mRS above the median at 24 months as an outcome. Only mRS at peak predicted a worse mRS outcome at 24 months, with no significant interactions between the variables. CI = confidence interval; IT = immunotherapy; IVIG = intravenous immunoglobulin; mRS = modified Rankin scale; OR, odds ratio; PLEX = plasma exchange; SSA = steroid sparing agent.

Appendix S1.

## Data Availability

Data can be shared with researchers conducting ethically approved studies.
