# Supplementary material for: Immunotherapy‐Resistant Neuropathic Pain and Fatigue Predict Quality‐of‐Life in Contactin‐Associated Protein‐Like 2 Antibody Disease
Source: Ann Neurol. 2025 Jan 18;97(3):521–8. doi: 10.1002/ana.27177 (PMC11831874; doi:10.1002/ana.27177)
Supplement: Supplementary file 1 — Supplementary Table S1. List of unrelated cases excluded from the study. All cases were seen by an experienced consultant neurologist and their CASPR2‐antibody seropositivity was deemed to be irrelevant to the presenting clinical syndrome. CASPR2 = contactin‐associated protein‐like 2; CSF = cerebrospinal fluid; EMG = electromyography; LCBA = live cell‐based assay; MRI = magnetic resonance imaging; VGKC = voltage‐gated potassium channel. [file ANA-97-521-s002.docx]

| **Participant** | **Diagnosis** | **Description** | **Age** | **Sex** | **LCBA Titre** |
| --- | --- | --- | --- | --- | --- |
| **1** | Functional movement disorder | Distractible cheek and shoulder movements triggered by eating and anxiety. | 34 | F | 1600 |
| **2** | Small fibre neuropathy | Longstanding distal pain and sensory loss on background of cutaneous vasculitis and alcohol excess. Weakly positive VGKC and transiently positive CASPR2 antibodies. | 75 | M | 200 |
| **3** | Focal epilepsy | 8-year history of recurrent facial droop episodes with retained awareness and transient CASPR2 antibody seropositivity but CSF negative. | 69 | F | 800 |
| **4** | Focal epilepsy | 10-year history of focal aware seizures with hippocampal atrophy on MRI. Transiently positive CASPR2-antibody seropositivity. | 28 | M | 200 |
| **5** | Idiopathic muscle twitching | Leg, arm and facial twitching with associated anxiety and poor sleep. No evidence of hyperexcitability on EMG and improved without immunotherapy. | 43 | F | 800 |
| **6** | Anxiety with isolated amnestic syndrome | 1 year of fluctuating ‘brain fog’ and attentional deficit, CASPR2 antibodies negative in CSF. | 30 | F | 800 |
| **7** | Focal epilepsy with severe learning disability | Childhood-onset drug resistant epilepsy and learning disability likely genetic in aetiology. Weakly positive VGKC antibody. | 35 | F | 200 |
| **8** | Focal epilepsy with severe learning disability | Previous childhood-onset epilepsy and learning disability, likely genetic in aetiology. New onset behavioural disturbance. | 35 | F | 1600 |
