# Supplementary material for: Immunotherapy‐Resistant Neuropathic Pain and Fatigue Predict Quality‐of‐Life in Contactin‐Associated Protein‐Like 2 Antibody Disease
Source: Ann Neurol. 2025 Jan 18;97(3):521–8. doi: 10.1002/ana.27177 (PMC11831874; doi:10.1002/ana.27177)
Supplement: Supplementary file 2 — Supplementary Table S2. Causes of death during the study. Relationships to either CASPR2‐antibody encephalitis or the consequences of immunotherapy. CASPR2 = contactin‐associated protein‐like 2; IT = immunotherapy; IVIG = intravenous immunoglobulin; PLEX = plasma exchange; SUDEP = sudden unexplained death in epilepsy. [file ANA-97-521-s001.docx]

| **Patient** | **Cause of Death** | **Age at Death** | **Immunotherapy** | **Death likely related to encephalitis or IT** | |
| --- | --- | --- | --- | --- | --- |
| 1 | Ischaemic heart disease and cardiac failure | 81 | Yes | | No |
| 2 | Sudden unexplained death in epilepsy (SUDEP) | 77 | Yes | | Yes |
| 3 | Bronchopneumonia (IT as complicating factor) | 83 | Yes | | Yes |
| 4 | Sepsis due to ulcer from peripheral vascular disease | 81 | Yes | | No |
| 5 | Ischaemic heat disease and cardiac failure | 70 | Yes | | No |
| 6 | Unknown | 86 | Yes | | Unknown |
