# Supplementary material for: Immunotherapy‐Resistant Neuropathic Pain and Fatigue Predict Quality‐of‐Life in Contactin‐Associated Protein‐Like 2 Antibody Disease
Source: Ann Neurol. 2025 Jan 18;97(3):521–8. doi: 10.1002/ana.27177 (PMC11831874; doi:10.1002/ana.27177)
Supplement: Supplementary file 3 — Supplementary Table S3. List of R packages and corresponding citations. [file ANA-97-521-s007.docx]

| **Package** | **Citation** |
| --- | --- |
| circlize | Gu, Z. (2014) circlize implements and enhances circular visualization in R. Bioinformatics. |
| ComplexHeatmap | Gu, Z. (2016) Complex heatmaps reveal patterns and correlations in multidimensional genomic data. Bioinformatics. |
| data.table | Barrett T, Dowle M, Srinivasan A, Gorecki J, Chirico M, Hocking T (2024). _data.table: Extension of `data.frame`_. R package version 1.15.4, <https://CRAN.R-project.org/package=data.table>. |
| dplyr | Wickham H, François R, Henry L, Müller K, Vaughan D (2023). _dplyr: A Grammar of Data Manipulation_. R package version 1.1.4, <https://CRAN.R-project.org/package=dplyr>. |
| gridextra | Auguie B (2017). _gridExtra: Miscellaneous Functions for "Grid" Graphics_. R package version 2.3,<https://CRAN.R-project.org/package=gridExtra>. |
| gridtext | Wilke C, Wiernik B (2022). _gridtext: Improved Text Rendering Support for 'Grid' Graphics_. R package version 0.1.5, <https://CRAN.R-project.org/package=gridtext>. |
| GGally | Schloerke B, Cook D, Larmarange J, Briatte F, Marbach M, Thoen E, Elberg A, Crowley J (2024). _GGally:Extension to 'ggplot2'_. R package version 2.2.1, <https://CRAN.R-project.org/package=GGally>. |
| ggridges | Wilke C (2024). _ggridges: Ridgeline Plots in 'ggplot2'_. R package version 0.5.6, <https://CRAN.R-project.org/package=ggridges>. |
| ggplot2 | H. Wickham. ggplot2: Elegant Graphics for Data Analysis. Springer-Verlag New York, 2016. |
| ggpmisc | Aphalo P (2023). _ggpmisc: Miscellaneous Extensions to 'ggplot2'_. R package version 0.5.5, <https://CRAN.R-project.org/package=ggpmisc>. |
| ggpubr | Kassambara A (2023). _ggpubr: 'ggplot2' Based Publication Ready Plots_. R package version 0.6.0, <https://CRAN.R-project.org/package=ggpubr>. |
| ggstatsplot | Patil, I. (2021). Visualizations with statistical details: The 'ggstatsplot' approach. Journal of Open Source Software, 6(61), 3167, doi:10.21105/joss.03167 |
| nptest | Helwig NE (2023). _nptest: Nonparametric Bootstrap and Permutation Tests_. R package version 1.1,<https://CRAN.R-project.org/package=nptest>. |
| patchwork | Pedersen T (2024). _patchwork: The Composer of Plots_. R package version 1.2.0,<https://CRAN.R-project.org/package=patchwork>. |
| questionr | Barnier J, Briatte F, Larmarange J (2023). _questionr: Functions to Make Surveys Processing Easier_. R package version 0.7.8, <https://CRAN.R-project.org/package=questionr>. |
| pheatmap | Kolde R (2019). _pheatmap: Pretty Heatmaps_. R package version 1.0.12, <https://CRAN.R-project.org/package=pheatmap>. |
| RColorBrewer | Neuwirth E (2022). _RColorBrewer: ColorBrewer Palettes_. R package version 1.1-3, <https://CRAN.R-project.org/package=RColorBrewer>. |
| Reshape | H. Wickham. Reshaping data with the reshape package. Journal of Statistical Software, 21(12), 2007. |
| Rstatix | Kassambara A (2023). _rstatix: Pipe-Friendly Framework for Basic Statistical Tests_. R package version 0.7.2,<https://CRAN.R-project.org/package=rstatix>. |
| tidyverse | Wickham H, Averick M, Bryan J, Chang W, McGowan LD, François R, Grolemund G, Hayes A, Henry L, Hester J, Kuhn M, Pedersen TL, Miller E, Bache SM, Müller K, Ooms J, Robinson D, Seidel DP, Spinu V, Takahashi K, Vaughan D, Wilke C, Woo K, Yutani H (2019). “Welcome to the tidyverse.” _Journal of Open Source Software_, *4*(43) 1686. doi:10.21105/joss.01686 <https://doi.org/10.21105/joss.01686>. |
| tidyr | Wickham H, Vaughan D, Girlich M (2024). _tidyr: Tidy Messy Data_. R package version 1.3.1,<https://CRAN.R-project.org/package=tidyr>. |
| viridis | Simon Garnier, Noam Ross, Robert Rudis, Antônio P. Camargo, Marco Sciaini, and Cédric Scherer (2024).viridis(Lite) - Colorblind-Friendly Color Maps for R. viridis package version 0.6.5. |
