# Supplementary material for: Immunotherapy‐Resistant Neuropathic Pain and Fatigue Predict Quality‐of‐Life in Contactin‐Associated Protein‐Like 2 Antibody Disease
Source: Ann Neurol. 2025 Jan 18;97(3):521–8. doi: 10.1002/ana.27177 (PMC11831874; doi:10.1002/ana.27177)
Supplement: Supplementary file 4 — Supplementary Table S4. Numbers of relapses and absolute risk for (A) first relapse and (B) all relapses, as in some cases patients had more than one relapse. Relapses were most frequent in the first 24 months from the peak of the disease and were more common in those with fatigue at onset than those without. [file ANA-97-521-s008.docx]

| **First Relapse** | | | | | | | | | |
| --- | --- | --- | --- | --- | --- | --- | --- | --- | --- |
|  |  | All Time Points | Peak to 6 months | 6 - 12 months | 12 - 24 months | 24 - 36 months | 36 - 48 months | 48 - 60 months | 60 - 72 months |
| No Fatigue |  | 3 | 1 | 0 | 1 | 0 | 1 | 0 | 0 |
| Fatigue |  | 9 | 3 | 3 | 3 | 0 | 0 | 0 | 0 |

A

A

| Absolute Risk (First Relapse) | | | | | | | | | |
| --- | --- | --- | --- | --- | --- | --- | --- | --- | --- |
|  |  | All Time Points | Peak to 6 months | 6 - 12 months | 12 - 24 months | 24 - 36 months | 36 - 48 months | 48 - 60 months | 60 - 72 months |
| No Fatigue |  | 0.08 | 0.03 | 0.00 | 0.03 | 0.00 | 0.04 | 0.00 | 0.00 |
| Fatigue |  | 0.24 | 0.08 | 0.09 | 0.10 | 0.00 | 0.00 | 0.00 | 0.00 |

B

| **All Relapses** | | | | | | | | | |
| --- | --- | --- | --- | --- | --- | --- | --- | --- | --- |
|  |  | All Time Points | Peak to 6 months | 6 - 12 months | 12 - 24 months | 24 - 36 months | 36 - 48 months | 48 - 60 months | 60 - 72 months |
| No Fatigue |  | 4 | 1 | 0 | 1 | 1 | 1 | 0 | 0 |
| Fatigue |  | 15 | 4 | 4 | 5 | 0 | 1 | 1 | 0 |

| Absolute Risk (All Relapses) | | | | | | | | | |
| --- | --- | --- | --- | --- | --- | --- | --- | --- | --- |
|  |  | All Time Points | Peak to 6 months | 6 - 12 months | 12 - 24 months | 24 - 36 months | 36 - 48 months | 48 - 60 months | 60 - 72 months |
| No Fatigue |  | 0.11 | 0.03 | 0.00 | 0.03 | 0.03 | 0.04 | 0.00 | 0.00 |
| Fatigue |  | 0.39 | 0.11 | 0.13 | 0.16 | 0.00 | 0.06 | 0.07 | 0.00 |
