# Supplementary material for: Immunotherapy‐Resistant Neuropathic Pain and Fatigue Predict Quality‐of‐Life in Contactin‐Associated Protein‐Like 2 Antibody Disease
Source: Ann Neurol. 2025 Jan 18;97(3):521–8. doi: 10.1002/ana.27177 (PMC11831874; doi:10.1002/ana.27177)
Supplement: Supplementary file 9 — Appendix S1. [file ANA-97-521-s006.docx]

**Supplementary Material**

**Methods**

*Flow cytometry testing of CASPR2-IgG*

Plasma or serum was tested by LCBA using visual observation (and end-point dilutions), and by flow cytometry, with live transfected human embryonic kidney (HEK) 293T cells incubated with patient serum, followed by a secondary antibody to IgG-Fc, IgG1 or IgG4. Mean fluorescence intensity (MFIs) were generated, as reported previously for LGI1-IgG.^14^
